# Magnetic gas sensing: working principles and recent developments

**DOI:** 10.1039/d0na00826e

**Published:** 2021-01-23

**Authors:** Pratik V. Shinde, Chandra Sekhar Rout

**Affiliations:** Centre for Nano and Material Sciences, Jain University Jain Global Campus, Jakkasandra, Ramanagaram Bangalore 562112 India r.chandrasekhar@jainuniversity.ac.in csrout@gmail.com

## Abstract

Gas sensors work on the principle of transforming the gas adsorption effects on the surface of the active material into a detectable signal in terms of its changed electrical, optical, thermal, mechanical, magnetic (magnetization and spin), and piezoelectric properties. In magnetic gas sensors, the change in the magnetic properties of the active materials is measured by one of the approaches such as Hall effect, magnetization, spin orientation, ferromagnetic resonance, magneto-optical Kerr effect, and magneto-static wave oscillation effect. The disadvantages of different types of gas sensors include their chemical selectivity and sensitivity to humidity and high-temperature operation. For example, in the case of chemiresistive-type gas sensors, the change in the sensor resistance can drastically vary in the real environment due to the presence of other gas species and the overall electrical effect is quite complex due to simultaneous surface reactions. Further, it is not easy to make stable contacts for powdered samples for the conventional electrical property-based gas sensors. Fire hazard is another issue for the electrical property-based hydrogen gas sensors due to their flammable nature at higher operating temperature. In this regard, to solve these issues, magnetic gas sensor concepts have emerged, in which the magnetic properties of the materials get modified when exposed to gas molecules. In this review article, the working principles, fundamentals, recent developments, and future perspectives in magnetic gas sensors are reviewed. Finally, the prospects and opportunities in these exciting fields are also commented upon based on their current progress.

## Abstract 2

In magnetic gas sensors, the change in the magnetic properties of the active materials is measured. In this review article, the working principles, fundamentals, recent developments, and future perspectives in magnetic gas sensors are reviewed.

## Graphical abstract

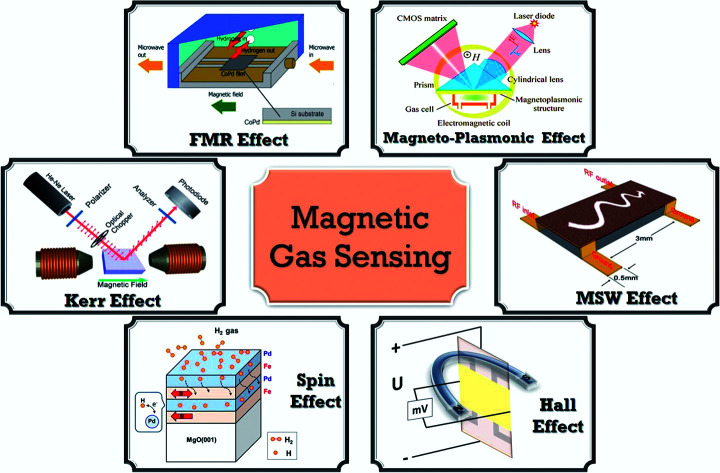

## 1 Introduction

A gas-sensing system or electronic nose can qualitatively or quantificationally detect specific gases, which is important in various applications such as industrial pollutant gas leakage detection (*e.g.*, H_2_, NO_2_, NH_3_, H_2_S, CO, and SO_2_), environmental monitoring, medical care, food industry, and homeland security.^1–7^ The performance of an ideal gas sensor is evaluated by its high responsivity, fast response/recovery times, great stability/repeatability, good selectivity, room-working temperature, low cost, and easy fabrication for practical applications.^1–7^ Gas sensors work on the principle of transforming gas adsorption on the surface of the active material into a detectable signal in terms of its changed electrical, optical thermal, mechanical, magnetic (magnetization and spin), and piezoelectric properties. Depending on these principles, many types of gas sensors with different transductions forms are developed, which include chemiresistors, field-effect transistors (FETs), Schottky and junction diode sensors, solid-state electrochemical sensors (SSES), quartz crystal microbalance (QCM), gas capacitors, surface acoustic wave (SAW), and plasmonic and surface-enhanced Raman spectroscopy (SERS) sensors. In chemiresistive-type gas sensor devices, the change in the system resistance or conductance due to gas adsorption is measured.^1–7^ In magnetic gas sensors, the change in the magnetic properties of the active materials is measured by one of the approaches such as Hall effect, magnetization, spin orientation, ferromagnetic resonance, magneto-optical Kerr effect, and magneto-static wave oscillation effect.^8,9^ Surface acoustic wave gas sensing utilizes the advantage of the piezoelectric effect of the sensor material, whose frequency gets shifted by the adsorption of analyte gas molecules.^10–13^ Optical gas sensors detect the gas species by monitoring their optical properties such as optical absorption, transmission, refractive index, and surface plasmon effects due to the interaction of the gas molecules with the active materials.^11–13^ New opportunities for sensing devices involve hot topics such as the internet of things (IoT) devices, wearable, flexible, and self-powered devices. Hence, research activities in these areas will lead to the fabrication of low cost, low power, small size, long term stability, and selective gas sensors for the detection of dangerous gas species.^11–14^

The disadvantages of different types of gas sensors include their chemical selectivity and sensitivity to humidity and high-temperature operation. For example, in the case of chemiresistive-type gas sensors, the change in the sensor resistance can drastically vary in the real environment due to the presence of other gas species and the overall electrical effect is quite complex due to simultaneous surface reactions.^11–14^ Further, it is not easy to make stable contacts for powdered samples for the conventional electrical property-based gas sensors. Fire hazard is another issue for the electrical property-based hydrogen gas sensors due to their flammability at higher operating temperature. In this regard, to solve these issues, magnetic gas sensor concepts have emerged, in which the magnetic properties of the materials get modified when exposed to gas molecules. These magnetic gas sensors are compatible with the processes involved in the fabrication of silicon devices and hence allows the integration of the sensors with on-chip devices.^15–17^ Systematic changes in the magnetic properties and exchange coupling, when exposed to gases, are monitored by different laboratory magnetometric techniques and equipment such as superconducting quantum interference devices (SQUID), vibrating sample magnetometers (VSM), polarized neutron reflectivity, X-ray resonant magnetic scattering (XRMS), Hall effect, optical Kerr effect, and ferromagnetic resonance setups.^15–17^ Moreover, nanomaterials also play a significant role in the further improvement of the gas sensor's performance. The nanomaterials show exceptional physical, chemical, as well as optoelectronic properties compared to their bulk counterparts.^18–22^ The high sensing performance comes from their high surface area and controlled grain size. Both these parameters expose more surface volume ratio for the gas sensing process. Their high surface area and controlled grain size play a significant role in achieving high sensing performance. In this review article, the working principles, fundamentals, and recent developments in magnetic gas sensors-based on different materials are reviewed. Furthermore, the future perspectives of magnetic gas sensors are discussed in the conclusion section.

## 2 Overview, theories, working principles, and recent developments of magnetic gas sensing

**Fig. 1:**
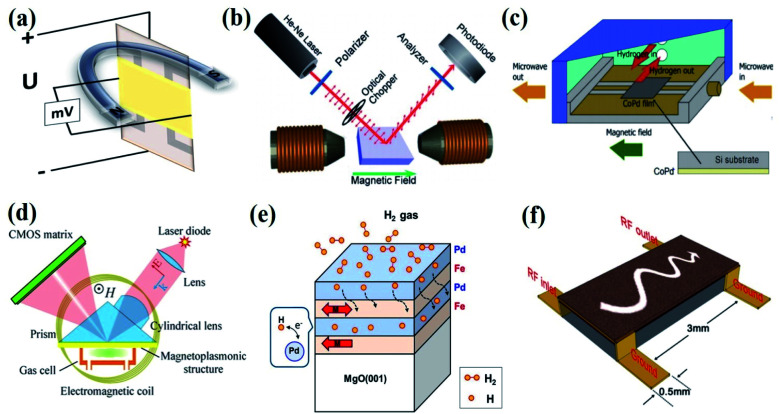
Magnetic effects used for the gas sensing device fabrication. (a) Hall effect, (b) Kerr effect. Reprinted with permission from ref. 22. Copyright (2016) Royal Society of Chemistry. (c) Ferromagnetic resonance (FMR) effect. Reprinted with permission from ref. 23. Copyright (2019) Elsevier. (d) Magneto-plasmonic effect. Reprinted with permission from ref. 24. Copyright (2016) Springer Nature. (e) Magnetic moment or spin effect. Reprinted with permission from ref. 25. Copyright (2018) Springer Nature. (f) Magnetostatic spin-wave (MSW) effect. Reprinted with permission from ref. 26. Copyright (2015) Royal Society of Chemistry.

Several effects of magnetism such as the Hall effect, Kerr effect, magnetization, spin change effects, the ferromagnetic resonance (FMR) effect, magneto-plasmonic effect, and magneto-static spin-wave (MSW) effect are employed for magnetic gas sensing applications (Fig. 1).^22–26^ Powder, thin films, and nanomaterials with magnetic (ferro- and antiferromagnets), diluted magnetic semiconducting (DMS) properties, and Pd alloys with transition metals (Co, Ni, Fe, Mn, and Cu) have been employed for magnetic gas sensing applications using different magnetic effects. Compared to their electrical property-based gas sensor counterparts, magnetic gas sensors have emerged as the more attractive candidate due to the following reasons: (i) no electrical contacts are needed to detect the gas, which lowers the risk of explosion due to fire when used in hydrogen-powered vehicles or in the presence of reactive chemicals or pollutants, (ii) magnetic response is much faster compared to chemiresistive sensors, (iii) the working temperature of the sensors can be room-temperature and can be tuned to a very low or very high temperature by choosing magnetic materials with different Curie temperatures (*T*_c_).^27–30^

### 2.1 Magnetization change-based magnetic gas sensors

#### 2.1.1 Overview, theories, and working principles

**Fig. 2:**
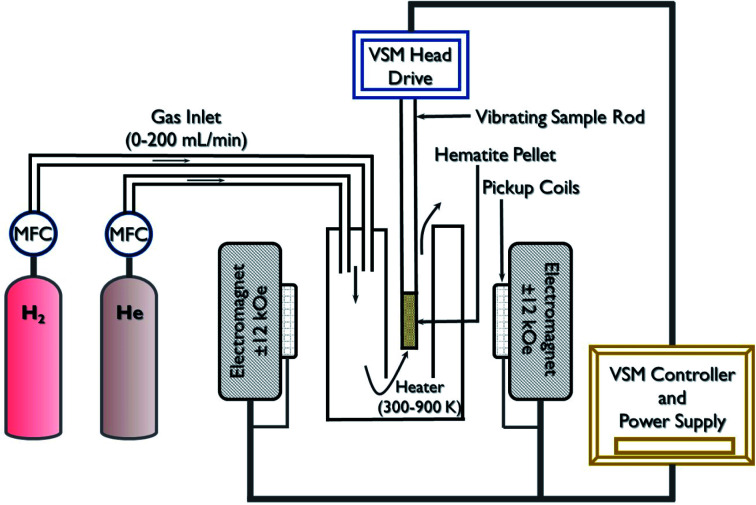
Schematic of the experimental set-up of the magnetization change-based magnetic gas sensor.

Metal oxide-based materials are known for their gas sensing applications since their magnetic parameters such as *M*_s_ (saturation magnetization), *M*_r_ (remanence magnetization), and *H*_c_ (coercivity) are very sensitive to reducing or oxidizing gases.^29–31^ A schematic illustration of the experimental set-up employed for magnetic gas sensing using the change in the magnetic properties is shown in Fig. 2. Usually, the set-up consists of a VSM capable of producing variable magnetic field *H* and accessories for gas flow arrangements. High-temperature heating units and mass flow controllers connected to gas cylinders are arranged for controlled sensing measurements in the presence of the gases at a different temperatures under the application of a magnetic field. When the semiconducting magnetic oxides or magnetic materials interact with the gas molecules (reducing or oxidizing), the surface absorption of ions/molecules leads to a change in the vacancy and defect concentration of the materials, which plays a crucial role in the change in *M*_s_, *M*_r_, and *H*_c_.^30^ The surface-adsorbed ions or molecules change the electronic structure at the surface, leading to a change in the overall magnetic behavior of the material due to the increase in the uncompensated surface spins.

#### 2.1.2 Recent developments

Punnoose *et al.*^29^ reported the magnetic hydrogen sensing properties of dilute magnetic semiconductor Sn_0.95_Fe_0.05_O_2_ nanoparticles. The systematic change in *M*_s_, *M*_r_, and *H*_c_ of Sn_0.95_Fe_0.05_O_2_ in the presence of H_2_ gas above 475 K demonstrated the experimental evidence of the concept of magnetic gas sensing. It was observed that for a temperature < 475 K, the chemisorbed water present on the nanoparticle surface inhibits particle–gas interaction, whereas for temperature > 475 K, the chemical reduction of the gas molecules by the oxygen species on the surface resulted in an increase in the carrier concentration. Further, the Sn_0.95_Fe_0.05_O_2_ nanoparticles showed stable ferromagnetic behavior after successive gas-sensing cycles, suggesting their good stability and long cycling performance. Saturation magnetization and remanence of the nanoscale antiferromagnetic haematite showed an increase of one to two orders of magnitude in the presence of H_2_ gas at concentrations in the range of 1–10% at 575 K.^30^ The *M*_s_ increased from 0.30 to 18 emu per g and *M*_r_ increased from 0.10 to 5 emu per g with a varied H_2_ gas (10%) flow from 0 to 200 mL min^−1^. Similarly, *H*_c_ increased from 60 to 250 Oe for 60 mL min^−1^ whereas no further change was observed with increased gas flow. X-ray photoelectron spectroscopy (XPS) analysis of the Fe_2_O_3_ samples before and after hydrogen treatment suggested that the observed change in the magnetization is due to the creation of oxygen vacancies. Density functional calculations of the MnN_4_ moiety embedded graphene (MnN_4_–Gp) upon interaction with gas molecules (CO, CO_2_, NO, NO_2_, N_2_O, SO_2_, NH_3_, H_2_O, H_2_S, CH_4_, O_2_, H_2_, and N_2_) showed obvious changes in its electronic and magnetic properties.^32^ The magnetic moment of MnN_4_–Gp decreased from 3.01 *μ*_B_ to 0.13, 1.01, and 2.01 *μ*_B_ after NO, CO, and NO_2_ absorption with recovery times of 2.5 × 10^14^ s, 1.4 s, and 8275 s, respectively.

**Fig. 3:**
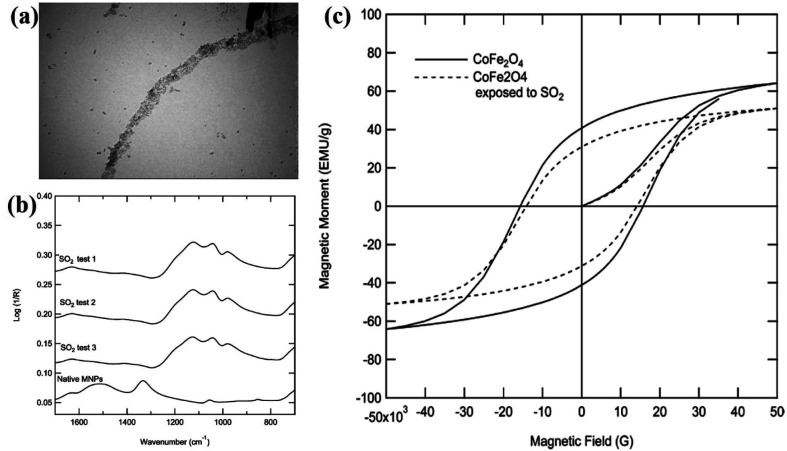
(a) TEM images of CoFe_2_O_4_ MNPs. (b) FTIR spectra of the native MNPs before exposure to sulfur dioxide as well as MNPs after exposure (test 1) and MNPs after exposure but without desorption (tests 2 and 3). (c) Magnetic susceptibility curves for MNPs both before and after sulfur dioxide breakthrough experiments. Reprinted with permission from ref. 33. Copyright (2012) American Chemical Society.

Glover *et al.*^33^ showed the adsorption of sulfur dioxide by CoFe_2_O_4_ nanoparticles and the corresponding changes in magnetism. Sulfur dioxide forms a sulfate upon adsorption on the particle surface by the chemisorption mechanism. Fig. 3a shows (transmission electron microscopy) the TEM images of CoFe_2_O_4_ magnetic nanoparticles (MNPs). Fig. 3b showing the Fourier transform infrared (FTIR) spectra of the native MNPs before exposure to sulfur dioxide as well as MNPs after exposure (test 1) and MNPs after exposure but without desorption (tests 2 and 3). The spectra of all the three tests are nearly identical, signifying that sulfur dioxide is retained on the surface of the MNPs. The magnetic susceptibility curves for MNPs samples before and after sulfur dioxide adsorption experiments are shown in Fig. 3c. As compared to the unexposed MNPs, the saturation magnetization decreases by 20%, the remnant magnetization decreases by 23%, and the coercivity decreases by 9% for CoFe_2_O_4_ MNPs after sulfur dioxide adsorption. The changes induced by gas adsorption are attributed to the magnetic metal cations at the surface layer of the nanoparticles that are coordinated with sulfur dioxide, which reduces the spin-orbital coupling and surface anisotropy.

The saturation magnetization and perpendicular anisotropy energy (*K*_p_) of Co/Pd multi-layered films are reported to change the reversibility with H_2_ gas adsorption and desorption.^34–38^ The change in *M*_s_ and magnetic anisotropy are attributed to electronic transfer to the Pd band near the Co/Pd interfaces when H_2_ is adsorbed. Electron transfer from Pd to the transition metal lowered the difference between the density of states of the spin-up and spin-down electrons at the Fermi level, leading to a net change in its magnetic properties.^36–38^ Similarly, magnetic coupling in Fe/Nb and Fe/V multi-layered films can be changed by hydrogen absorption, which was confirmed by SQUID magnetization measurements.^39–41^ Though the method is effective, the high cost and tricky optimization of several setup parameters limit its application.

### 2.2 Hall effect-based magnetic gas sensors

#### 2.2.1 Overview, theories, and working principles

Hall effect is the production of a voltage difference known as “Hall voltage” across an electrical conductor transverse to an electron current flowing through the conductor and to an applied magnetic field perpendicular to the current. Hall effect is classified into ordinary Hall effect (OHE) and extraordinary Hall effect (EHE) depending on its origin in magnetic materials. Due to their advantageous properties, both OHE and EHE concepts are employed for the fabrication of magnetic gas sensors.^15,42,43^ In the OHE approach, the change in the Hall voltage with and without target gas is measured.^43^ The sensor responses for oxidizing and reducing gases are defined as:

For oxidizing gas,

1 *S*_0_ = (*V*_g_ − *V*_a_)/*V*_a_

For reducing gas,

2 *S*_0_ = (*V*_a_ − *V*_g_)/*V*_g_

Where *V*_a_ and *V*_g_ are the Hall voltages in air and in the target gas, respectively. If only the OHE is considered, the Hall voltage due to the Lorentz force acting on the moving charge carriers is defined by eqn (3)–(5).^43^

3 *V*_H_ = *R*_H_ × (*I*_*x*_ × *B*_*z*_)/*d*_*y*_

Since for the semiconductor, the Hall resistance,

4 *R*_H_ = 1/*e* × [(*p* − *nb*^2^)/(*p* + *nb*)^2^]

with *b* = *μ*_*n*_/*μ*_*p*_where *n* and *p* are electron and hole concentration, *μ*_*n*_ and *μ*_*p*_ are the electron and hole mobility, respectively, and *e* is the elemental charge (electron).

From the law of mass action, *p* = *n*_i_^2^/*n*, where *n*_i_ is the electron and hole concentration in an undoped semiconductor material. Using a normalized electron concentration, *x* = *n*/*n*_i_5
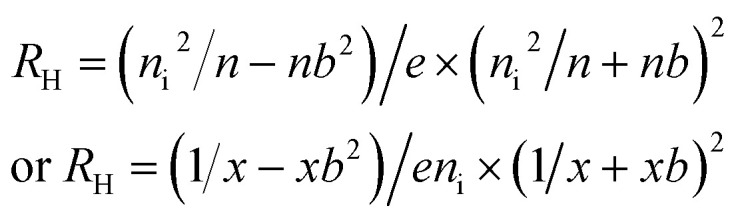


By using these relations, the Hall co-efficient for the active sensor materials in the presence and absence of the gases can be evaluated.

Gerber *et al.*^15^ employed the EHE effect for magnetic H_2_ gas sensing applications of the Pd/Co film as the sensor material. By following their approach, the electric current flowing along the magnetic film generates a voltage in the direction perpendicular to the current direction.

6 *V*_H_ = *R*_H_*I* = *I*/*t*(*R*_OHE_*B* + *R*_EHE_*μ*_0_*M*)

Here, *I* is the current, *t* is the thickness of the film, and *B* and *M* are components of magnetic induction and magnetization perpendicular to the film, respectively.

*R*_OHE_ is the ordinary Hall effect coefficient related to the Lorentz force acting on moving charge carriers and *R*_EHE_ is the extraordinary Hall effect coefficient associated with a break in the right-left symmetry at spin–orbit scattering in magnetic materials. Since the EHE term is significantly high, *i.e.*, *R*_EHE_ ≫ *R*_OHE_,

7 *R*_H_ = *V*_H_/*I* = *μ*_0_*R*_EHE_*M*/*t*

which implies that the Hall signal *μ*_0_*M* with Hall resistivity, and *ρ* ∼ *R*_H_/*t* and 
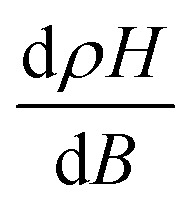
 indicate the dominance of the right hand over the left-hand spin-orbital scattering.

The Co/Pd films rich in Co exhibited +ve polarity, whereas the Pd-rich sample exhibited −ve polarity. The sensitivity of the magnetic gas sensors based on the EHE contribution can be calculated by the formula8
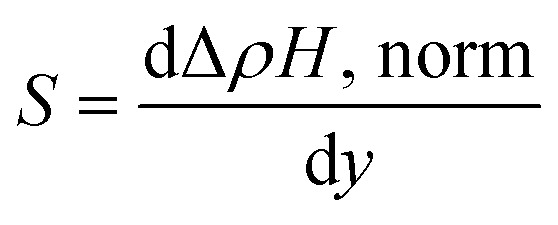
where

9 Δ*R*_H_, norm(*y*) = Δ*R*_H_(*y*)/*R*_H_(0) = [*R*_H_(*y*) − *R*_H_(0)]/*R*_H_(0)

**Fig. 4:**
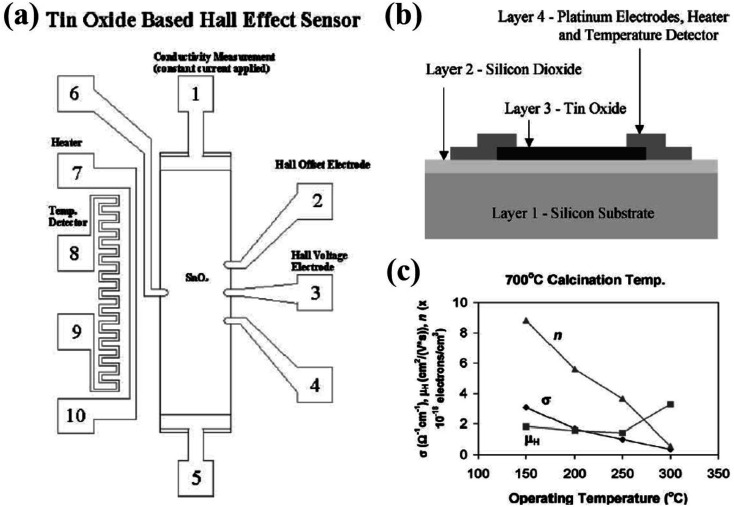
(a) Tin-oxide based Hall effect sensor. (b) Cross-sectional view of the tin-oxide based Hall effect sensor. (c) Tin oxide thin film conductivity (*σ*), electron density (*η*), and Hall mobility (*μ*_H_) in air *vs.* operating temperature for sensor calcined at 700 °C. Reprinted with permission from ref. 44. Copyright (2001) Elsevier.

Hammond *et al.*^44^ designed a tin-oxide-based sensor, *viz.*, the Hall effect sensor as shown in Fig. 4a and its cross-sectional view is presented in Fig. 4b. The sensor is made up of four layers, wherein the bottom silicon layer (0.3 mm thick) served as a support for the remaining layers. The insulator layer of silicon dioxide between the silicon substrate and the remaining sensor component is nearly 1 μm thick. The third rectangular layer of tin oxide (0.5 mm × 2.0 mm × 1100 Å) shows good adhesion to the SiO_2_ surface during deposition and operation. The fourth layer was made up of 3000 Å-thick platinum, consisting of electrodes, in order to measure the conductivity, temperature, and hall voltage. The Hall effect is the induction of transverse voltage in a current-carrying conductor when the conductor is exposed to the magnetic field. Here, the magnetic field is applied perpendicular to the tin oxide surface using a 5403 electromagnet system. The Hall voltage (*V*_H_) was measured before exposure to H_2_ in air and after the sensor reached the maximum conductivity level when exposed to H_2_ in air. The values for conductivity (*σ*), electron density (*η*), and Hall mobility (*μ*_H_) in air as a function of the operating temperature are shown in Fig. 4c. The Hall mobility remains stable compared to the variation in the conductivity and electron density. All the films have Hall mobility value in the range of 0.9 cm^2^ V^−1^ s^−1^ to 1.83 cm^2^ V^−1^ s^−1^. The experimental results indicated that the Hall mobility sensitivity slightly increased with the operating temperature and weakly depends on the H_2_ concentration in air.

#### 2.2.2 Recent developments

Undoped and doped metal oxide films based on Hall effects are reported for selective and high-performance magnetic gas sensing applications.^21,43,45^ Lin *et al.*^43^ reported NO_2_ sensing properties of WO_3_ films based on the ordinary Hall effect. The sensor displayed a selective response of 3.27 for 40 ppm NO_2_ with the response and recovery times of 36 s and 45 s, respectively, based on the sensor working principles described in eqn (1)–(5). The output Hall voltage increased for oxidizing gas such as NO_2_, whereas it decreased for reducing gases such as NH_3_ and H_2_S. The surface adsorbed oxygen ion species (O_2_^−^ and O^−^) played a crucial role in the enhanced magnetic NO_2_ sensing performance of WO_3_.^46^ Due to the n-type semiconducting nature of WO_3_ (with *n* > *n*_i_) and the presence of surface-adsorbed oxygen species, the following NO_2_ sensing mechanisms are expected to occur.

10 NO_2(gas)_ + e^−^ → NO_2(ads)_^−^

11 NO_2(gas)_ + O_2(ads)_^−^ + 2e^−^ → NO_2(ads)_^−^ + 2O_(ads)_^−^

Due to these surface reactions, the electron concentration on the surface of WO_3_ decreases when the electron concentration is greater than 1.14*n*_i_. This process helped to increase the Hall coefficient, correspondingly increasing the Hall voltage in the presence of NO_2_ gas.^43^ Based on similar working principles, the SnO_2_ nanowires-based Hall sensor showed good H_2_ sensing properties with a response of 7.81 towards 1000 ppm H_2_ at 125 °C.^21^ From the experimental observations, it has been demonstrated that the Hall coefficient of the gas sensor devices was dependent on the gas concentration and the high surface area of the nanostructures helped to achieve enhanced gas-sensing performance.

**Fig. 5:**
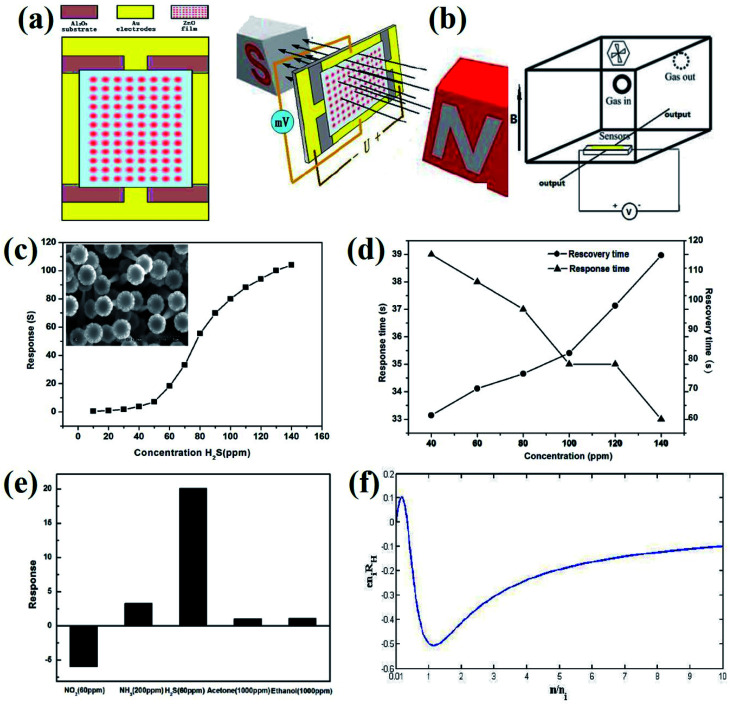
(a) Schematic of the sensor. (b) Measurement system for testing gas sensors. (c) Response of the Hall effect sensor to H_2_S with varied concentration of 10–140 ppm. Inset: Scanning electron microscopic image of the ZnO sample. (d) The response and recovery time of the Hall effect sensor to various concentrations of H_2_S. (e) The response of the Hall effect sensor to various gases. (f) Normalized Hall coefficient *vs.* normalized electron concentration. Reprinted with permission from ref. 47. Copyright (2017) ESG.

Lin *et al.*^47^ reported the detection of H_2_S at room temperature using ZnO sensors based on the Hall effect. The electrical and sensing characteristics of the sensors (Fig. 5a) were detected through the bench system under ambient temperature, as shown in Fig. 5b. The sensor was placed in a magnetic field perpendicular to the current, which is moving through the sensor. A constant voltage of 5 V was continuously supplied to the sensor circuit from a power supply and the Hall voltage of the sensor was measured using different concentrations of the gas. At room temperature, the response of the sensor increases exponentially along with increasing gas concentration (Fig. 5c). For 100 ppm H_2_S, the recovery and response time was 82 s and 35 s, respectively. As the concentration increased, the response time becomes shorter and the recovery time becomes longer (Fig. 5d). From Fig. 5e, it was observed that the Hall effect sensor displayed a negligible response to acetone and ethanol. Also, the sensor showed a higher response to 60 ppm H_2_S than that at 200 ppm NH_3_ and 60 ppm NO_2_. The behavior of the sensor based on the Hall effect to H_2_S is associated with a change in the electron concentration and has potential applications in actively detecting toxic gas without using any electrical power.

**Fig. 6:**
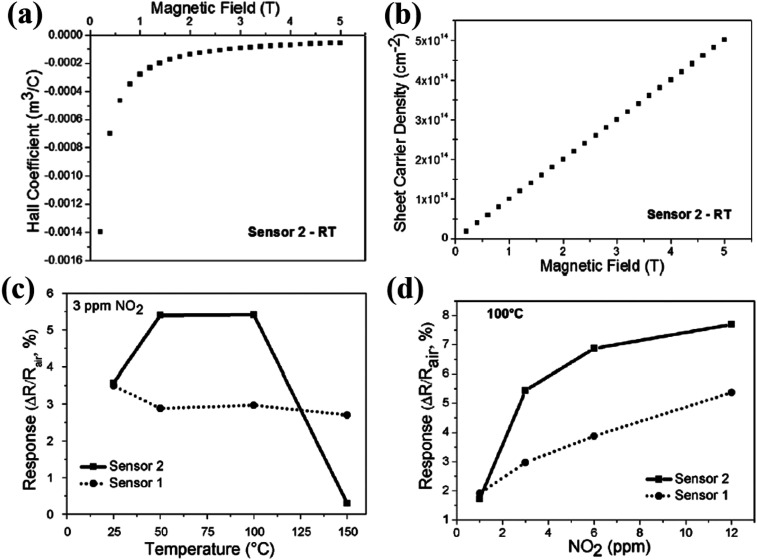
(a) Hall coefficient. (b) Sheet carrier density of the as-deposited galinstan film (Sensor 2, 10 μm thickness). (c) Response of the as-deposited sensors towards 3 ppm NO_2_ as the function of the operating temperature. (d) Response amplitude of the as-deposited sensors as a function of NO_2_ concentration. Reprinted with permission from ref. 48. Copyright (2017) Elsevier.

Shafiei and his group fabricated conductometric sensors based on an oxidized liquid metal galinstan layer and investigated their sensitivity towards NO_2_, NH_3_, and CH_4_ gases.^48^Fig. 6a and b indicates the negative Hall coefficients as a function of the magnetic field for Sensor 2. Also, a linear relationship between the sheet carrier density, ns, and magnetic field is given. Fig. 6c indicates the higher response of Sensor 2 than Sensor 1, which is attributed to better film coverage (as confirmed by scanning electron microscopy (SEM)) and a larger active surface area for gas interaction. A response of 7.7% and 5.4% was recorded for 12 ppm NO_2_ using Sensor 2 and Sensor 1, respectively (Fig. 6d). The Hall effect measurements revealed that the p-type oxide film influenced the sensing response towards different gases.

Extra-ordinary Hall phenomena (EHE, eqn (6)–(9)) are employed in ferromagnetic Co/Pd films for hydrogen gas sensing applications.^15,49^ Due to the high solubility of hydrogen in Pd, it is usually chosen to make stacked layers of Pd with the transition metal for H_2_ sensing. The palladium lattice expands significantly with the absorption of H_2_ (0.15% for α-phase and 3.4% for β-phase), which leads to the formation of palladium hydride; this phenomenon is usually employed for the fabrication of H_2_ sensors based on Co–Pd alloys and multi-layered films.^15,50–52^ Field dependence of the Hall resistivity of the Co_*x*_Pd_1−*x*_ film (0 ≤ *x* ≤ 0.4) displayed that the polarity reverses between *x* = 0.15 and *x* = 0.2. Samples with richer Co content were found to exhibit positive polarity while samples with richer Pd content showed negative polarity. Hall effect resistance as a function of the magnetic field for the Co_*x*_Pd_1−*x*_ films in H_2_ (4%) showed changes in the hysteresis loop area. The Hall effect (EHE) in the optimized sample (Co_0.17_Pd_0.83_) showed a sensitivity > 240% per 10^4^ ppm at H_2_ concentration below 0.5% in the hydrogen/nitrogen atmosphere, which was 2 orders of magnitude higher than that of the conductance-based sensor. From the detailed H_2_ sensing studies, it was demonstrated that depending on the film composition, thickness, and field of operation, enhanced EHE response of the Co/Pd films could be achieved and these types of materials emerge as an attractive candidate for magnetic gas sensing.^15,49^ Hall effect-based sensors depend on the operating temperature, p- or n-type conductivity of the material, as well as on the film composition, thickness, and field of operation.

### 2.3 Kerr effect-based magnetic gas sensors

#### 2.3.1 Overview, theories, and working principles

**Fig. 7:**
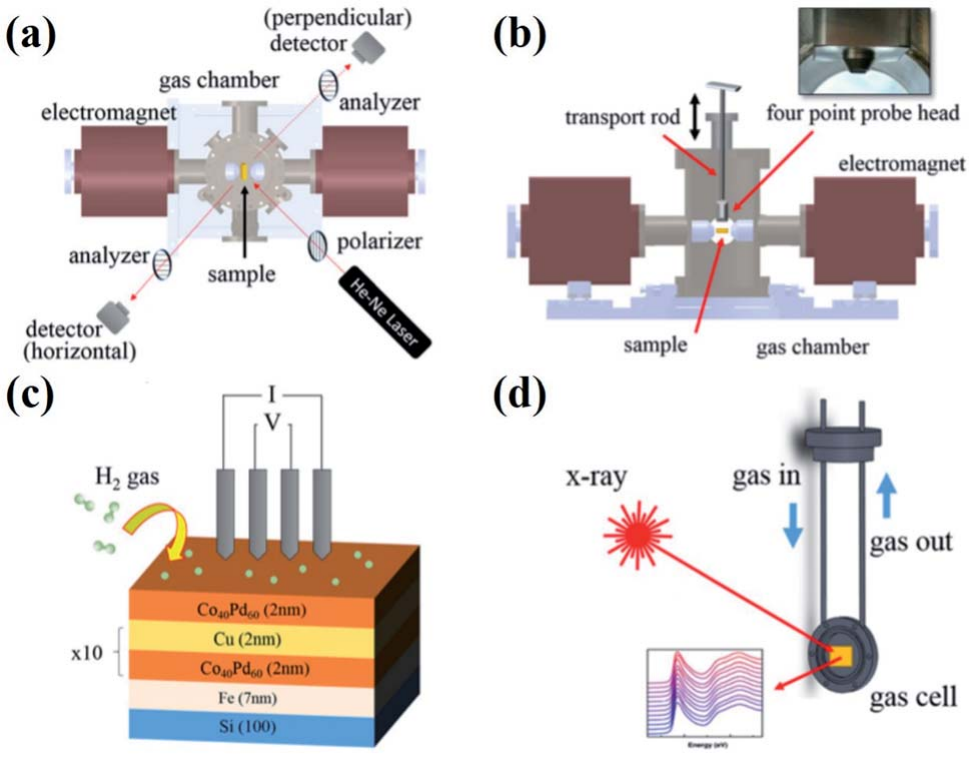
MOKE experimental set up used for magnetic gas sensing: (a) Top and (b) side views along with the four-probe electrical measurement set up. (c) Schematic illustration of the multi-layered films with respect to the four-probe measurements under H_2_ atmosphere. (d) Gas cell integrated with the *in situ* X-ray characterization. Reprinted with permission from ref. 16. Copyright (2017) American Institute of Physics.

Magneto-optic Kerr effect (MOKE), which is related to the change in the polarization and intensity of reflected light from a magnetized surface, is employed for gas sensing applications.^9,16,22,53,54^ This type of magnetic gas sensing measurement is carried out by a MOKE magnetometer with the application of a very small magnetic field (∼50 Oe) at room temperature. The MOKE technique offers great advantages due to its high surface-sensitive properties and the signal is not affected by the paramagnetic and diamagnetic contributions of the substrates. A typical MOKE experimental set up along with *in situ* X-ray analysis and four-point probe electrical measurement are shown in Fig. 7a–d.^16^ By this approach, the change in the reflected light beam and magnetic properties of the materials, *i.e.*, *M*_s_, *H*_c_, and squareness (*M*_r_/*M*_s_) of the hysteresis loop along with the perpendicular and horizontal (in-plane) directions are monitored by rotating the incident polarized light in the presence of the gas. The sensitivity of the magnetic sensor can be evaluated by^55^

12 *S* = (*B*_air_ − *B*_gas_)/*B*_gas_

Where magnetic induction *B* = *M*/*M*_s_.

The change in the Kerr intensity of the reflected light in positive and negative directions (+*m* and −m) as a parabolic function of the analyzer angle (*φ*) is represented by13
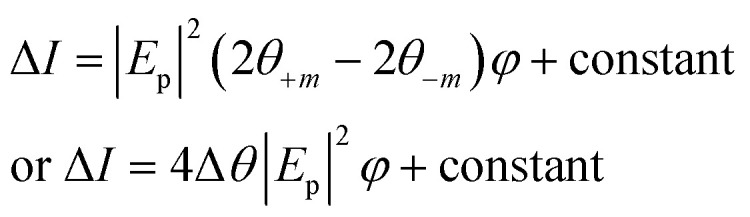
where *E*_p_ is the p component of the reflected light.

Also, the relative change in the Kerr rotation angle is defined by14
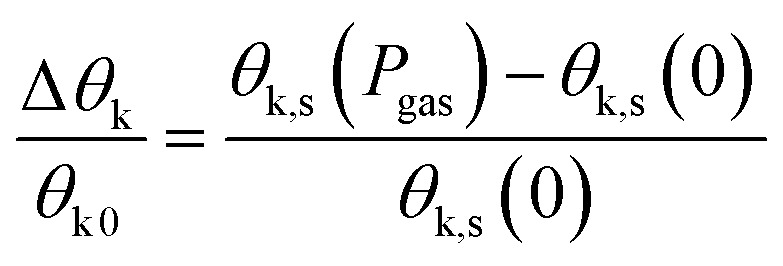
which is used to evaluate the magnetic gas sensing by MOKE, where *θ*_k,s_ is the saturation Kerr rotation signal in the gas. In some reports on MOKE-based gas sensors, it is demonstrated that the changes obey^54^15
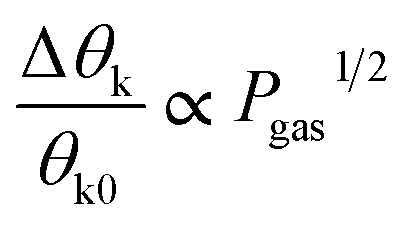


#### 2.3.2 Recent developments

**Fig. 8:**
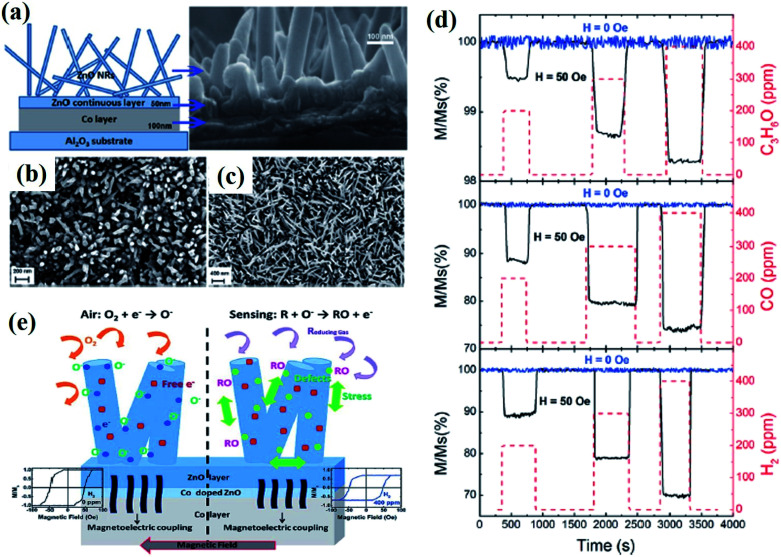
Magnetic gas sensing of the ZnO/Co nanorods by the MOKE magnetometer. (a) Schematic illustration and SEM images of the ZnO nanorods grown on Co-layer, SEM images of (b) MGS1 and (c) MGS2 ZnO nanorods. (d) Magnetization induced gas sensing performance of the MGS2 sample with different concentrations of C_3_H_5_O, CO, and H_2_ with (50 Oe: black) and without (blue lone) an applied magnetic field. (e) Schematic representation of the magnetic gas sensing process and an example of the hysteresis loops measured during the sensing process. Reprinted with permission from ref. 22. Copyright (2016) Royal Society of Chemistry.

ZnO nanorods arrays with different sizes grown on Co-layers have been utilized for magnetic gas sensing applications by MOKE.^22^ By the MOKE magnetometer, the change in the polarization of a linearly polarized light after reflection on the magnetized sample (ZnO:Co) surface is measured. For magnetic gas sensing, the signals are collected and monitored by an Si-photoanode continuously under a constant applied field of ∼50 Oe and in different concentrations of gases. Ciprian *et al.*^22^ reported the comparative magnetic gas sensing properties of the ZnO nanorod films with different sizes [MGS1: 100–200 nm long with irregular tips, MGS2: ∼1 mm long, average dia ∼ 20–30 nm] (Fig. 8). The sensor showed good sensitivity to all the tested gases even at the lowest concentration with a linear increase in the signal with an increase in the concentration. The sensitivity of the sensor is found to be greater than three times the standard deviation of the background noise and the detection limits are 10, 5, and 4 ppm C_3_H_6_O, CO, and H_2_, respectively. The sensors displayed response and recovery times less than 60 s with good stability and recyclability performance. The mechanism involved in magnetic gas sensing is summarized in Fig. 8e. It is demonstrated that the structural defects and oxygen vacancies in ZnO played an important role and enhanced the internal stress. The piezoelectric properties as well as the polarizability of the ZnO nanorods get modified, which resulted in the release of free electrons during the absorption of the gas molecules. These effects changed the magneto-elastic and magneto-electric contributions to the system anisotropy, transduced by Co, and led to a decrease in the magnetization. Manera *et al.*^56^ presented a novel combination of materials, namely, a magneto-plasmonic Au/Co/Au trilayer with TiO_2_ and its remarkable capabilities as a gas sensor by magneto-optical surface plasmon resonance measurements.

Pd/transition metal (Co, Fe, and Ni) bilayer, multilayer, and alloyed films have been investigated for magnetic gas sensing by the MOKE approach.^54,55,57,58^ Since upon gas exposure, the extinction angle of MOKE gets shifted and the Kerr intensity significantly increases, thus, by choosing the proper analyzer angle, enhanced sensing performance could be achieved. In a comparative MOKE study of Pd covered with Fe, Co, and Ni magnetic layers, it was demonstrated that the magneto-optical enhancement could reach up to 35–70% with hydrogen absorption depending on the magnetic layer. An enhancement of 40%, 35%, and 50–70% was obtained for Pd/Fe, Pd/Co, and Pd/Ni bilayers in 1 × 10^5^ Pa H_2_.^58^ For the Pd/Co/Pd trilayer film, hydrogenation not only increased the Kerr signal but also significantly enhanced the Hc by 17%.^59^ Lin *et al.*^55^ reported a more pronounced reduction in the magnetization in the presence of H_2_ gas for Co_14_Pd_86_ because of the large Pd content around the Co-atoms. Hydrogenation introduced structural defects due to the lattice expansion and contraction of hydrogen absorption and desorption. Upon exposure of H_2_ gas, the squareness of the hysteresis loop both in the perpendicular and in-plane directions showed a large transition from approximately 10% to 100%, and the saturation Kerr signal reduced to nearly 30% of the pristine value. The reversibility of transition from the hysteresis loops and the analysis of the change in the intensity of reflected light happened with a response time of ∼2–3 s. These observations indicated the formation of palladium hydride, which transformed the short-range coupled and disordered magnetic state of the Co_14_Pd_86_ film to a long-range ordered ferromagnetic state and induced a decrease in the magnetic moment. Further, the enhancement in long-range ordering and the reduction in the magnetic moment were attributed to the change in the electronic structure in the Co_14_Pd_86_ film due to hydrogen absorption. A similar type of observation is reported for the Co (0.5 nm)/Pd (3 nm) multilayer film.^60^ For a continuous film, *H*_c_ was enhanced by 47% and the Kerr intensity was significantly reduced to 10% of the pristine value after hydrogenation. For the nanodots, hydrogenation led to a 25% reduction in *H*_c_, whereas for nanodot chains, the shape of the magnetic hysteresis loop could be modulated. In another report, the MOKE effect was observed in magnetic hysteresis loops of the films with a Pd content of 61%, 76%, and 86%, whereas no observable changes were obtained in the MOKE hysteresis loops for Co-rich alloy films.^61–63^ For the Co_30_Pd_70_ alloy films, considerable hydrogen-induced reduction in the magnetic coercivity by a factor of 1/5 in the longitudinal direction and the enhancement in magnetic resonance to a saturation ratio from 60% to 100% were reported.^64,65^ For the Co_40_Pd_60_/Cu layered films, the *M*_s_ (induced resistance change) dropped (increased) by a factor of 5 in an H_2_ pressure of 75 kPa.^16^ In the case of perpendicularly-magnetized Pd/Co/Pd trilayers, the hydrogenation not only increased the Kerr signal but also significantly enhanced the Hc by 17%.^59^

**Fig. 9:**
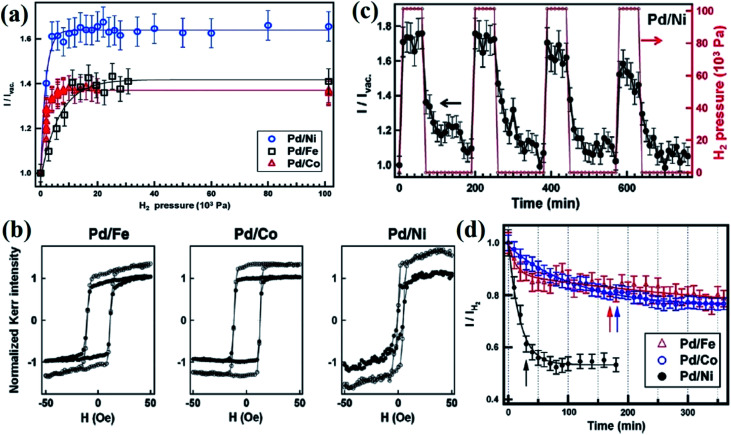
(a) Normalized Kerr intensity as a function of H_2_ pressure. The trend lines are included and do not represent the fitting curves. (b) The MOKE hysteresis loops measured in 1 × 10^5^ Pa H_2_ (open circles) and a vacuum (filled circles). (c) Normalized Kerr intensity (left axis) measured with cycles of H_2_ exposure (right axis) for the demonstration of reversibility. (d) Normalized Kerr intensity as a function of time, after H_2_ is pumped out. The trend lines are included and do not represent the fitting curves. The arrows indicate the time constant when the Kerr intensity recovered 80% of the enhancement. Reprinted with permission from (58). Copyright (2013) Elsevier.

Lin *et al.*^58^ reported on the reversible change of MOKE in the Pd-covered magnetic thin films by controlling the H_2_ absorption. This absorption induced reversible MOKE enhancement in Pd-covered Fe, Co, and Ni thin films. As shown in Fig. 9a, the Kerr intensity drastically increased with H_2_ exposure and saturated at ∼5 × 10^3^ Pa, while Pd/Co and Pd/Fe were saturated at ∼8 × 10^3^ and ∼11 × 10^3^ Pa, respectively. Fig. 9b indicates that H_2_ adsorption does not affect the magnetization processes of the magnetic films and the magneto-optical enhancement should originate from H_2_-induced change in the optical properties of Pd. Fig. 9c represents the reversibility of this H_2_ adsorption effect in the Pd/Ni bilayer. The Kerr intensity reached a saturation value within a few minutes and decreased slowly after the recovery of the vacuum. Fig. 9d indicates the time constant when the Kerr intensity recovered 80% of the H_2_-induced enhancement. These results indicate that the extinction angle of MOKE becomes shifted and correspondingly, the Kerr intensity is significantly increased after H_2_ exposure. This MOKE augmentation originates from the change in the optical properties of the hydrogenated Pd layer.

Hydrogenation-induced MOKE studies revealed that by tuning the thickness of the Pd layer in Pd/Fe bilayers, the MOKE extinction angle can be gradually shifted by 0.6%.^66^ The enhancement in the Kerr intensity reached 35–40% by exposure of 1 atm hydrogen with a different analyzer angle. Mudinepalli *et al.*^67^ investigated the annealing effect on the hydrogenation-related magnetic property changes of the Pd/Fe bilayer films. It was revealed that Pd–Fe inter diffusion, *i.e.*, alloy formation occurred at about 700 K and the *H*_c_ increased by 2–3 times with an annealing temperature up to 700 K. Hydrogenation effect was observed in the 710 K annealed sample with an increase in the in-plane *H*_c_ by 10% when the hydrogen pressure was above 200 mbar. With further annealing in the range of 740–800 K, the hydrogenation effect on *H*_c_ became nearly unobservable. Hydrogenation induced changes in the magnetic properties of the Fe–V nanoclusters and the bilayer films were investigated by the MOKE effect.^24,68,69^ It was revealed that the lattice constant and electronic properties of the vanadium-intermediated nanoclusters get modified by the hydrogen content, which directly influences the exchange coupling between the Fe nanostructures.^68^ Hsu *et al.*^25^ revealed that by selecting a suitable Pd thickness under the application of a magnetic field perpendicular to the easy axis of the bottom Fe layer, two well-splitted hysteresis loops with almost zero Kerr remanence can be obtained. The split of the hysteresis into two double loops is reported to be due to the 90° rotation of the top-Fe moment. By the exposure to hydrogen gas, the separation of the two minor loops was increased due to the formation of the Pd-hydride formed in the space layer. These investigations suggested that the Pd space layer mediated the magnetic interlayer coupling, which was sensitive to the hydrogen atmosphere, demonstrating Fe/Pd multilayers as an emerging material for a great magnetic resistance (GMR)-type sensor for H_2_ sensing.

### 2.4 FMR-based magnetic gas sensors

#### 2.4.1 Overview, theories, and working principles

**Fig. 10:**
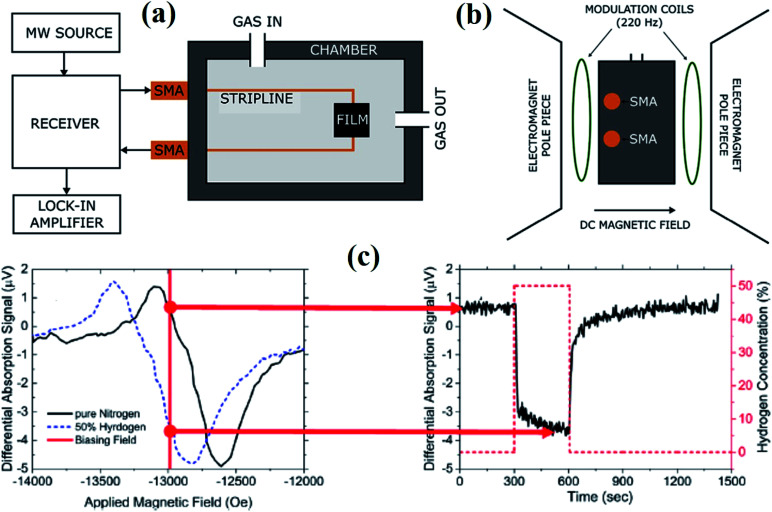
Schematic illustration of the (a) FMR based gas sensor set up. (b) The chamber during measurement mounted between the poles of the electromagnet and the modulation coils. (c) Typical FMR spectra in the presence of nitrogen and H_2_ gas used to evaluate the sensor response. Reprinted with permission from ref. 77. Copyright (2017) Elsevier.

Perpendicular magnetic anisotropy (PMA) is known to be induced at the interface between a ferromagnetic metal (FM) layer and a non-magnetic (NM) heavy metal such as Pt or Pd in an FM/NM bilayer or multilayer films.^17,70,71^ The origins of the interface PMA are identified to be due to the (i) breaking of the crystal symmetry at the interface, (ii) interface alloying, (iii) and the effect of magnetostriction.^72,73^ The first two effects are termed as electronic contribution due to their electronic nature. The third contribution is due to the indirect elastic strain at the interface on the magnetization of the FM layer due to the NM layer.^74^ This term due to PMA is usually termed as “magneto-elastic”. Several reports have demonstrated that the high frequency, resonant magnetization dynamics within the NM/FM interface such as Pd/Co films can be exploited for gas (H_2_) sensing.^17,70,71^ For example, the functionality of the Pd/Co film-based H_2_ sensor device prototype is based on a modification of the strength of PMA at the interface between Co and Pd layers upon adsorption of H_2_ by Pd.^75^ FMR represents an eigen excitation in the magnetic materials, which exists in the microwave range and is originated as the resonant absorption of microwave power by a magnetic sample when the frequency of the applied microwave source is equal to the resonance frequency of the material.^76^ For this type of a gas sensor device, FMR is used to measure the change in PMA. By this approach, the frequency of the input signal drives the FMR constant and in the presence of a gas, the FMR peak position for the Co-layer shifts to a lower applied field H, which is due to the decrease in the PMA in the presence of the analyte gas molecule. The magnitude of the FMR peak shift is used to determine the sensitivity of the sensor. The typical experimental set up used for the FMR-based gas sensor measurements is shown in Fig. 10.^77^ During the FMR measurement, the substrate with the sample is placed on top of the microstrip line with the sputtered film facing the microstrip. The radiofrequency signal transmitted through the microstrip line is measured to register the FMR absorption. A reduction in the transmitted power at a given frequency under the application of an external magnetic field signifies the functionality of the sensor device. To improve the signal-to-noise ratio, a field-modulated FMR method is employed, which is shown in Fig. 10b. A further enhancement in the signal-to-noise ratio is achieved by employing a microwave interferometric receiver, as shown in Fig. 10a. Typical field-resolved FMR traces obtained in pure nitrogen and H_2_ gas are shown in Fig. 10c, which are used for the analysis of the gas-sensing performance.

#### 2.4.2 Recent developments

By employing the FMR change effects, the PdCo bilayer or multi-layered films are employed for selective H_2_ sensing applications.^17,23,71,75^ Lueng *et al.*^23^ studied the FMR response of the Co_*x*_Pd_1−*x*_ alloy films (*x* = 0.65, 0.39, 0.24, and 0.14) in the presence of H_2_ gas. It was demonstrated that without any special processing, the films with *x* = 0.39 and 0.24 demonstrated promising H_2_ gas sensing properties in a very broad concentration range from 0.05% to 100%. For *x* = 0.24, a continuous film of Co_*x*_Pd_1−*x*_, showing a 1.7 times change in the FMR peak shift when the H_2_ concentration changed from 10% to 67%, was observed. Further, it is demonstrated that by properly adjusting the magnetic field, a wide range of H_2_ gas concentrations (0.2–100%) can be detected by the Pd/Co bilayer film with better sensing performance.^77^ Across this broad H_2_ concentration range, the sensitivity varied by 80 times from 0.45 μV %^−1^ (at low concentration) to 0.01 μV %^−1^ (for high concentration ∼100%) with no signal saturation. This reveals that a proper adjustment of the magnetic field is needed to achieve better sensing performance.

The observed decrease in the resonance linewidth upon hydrogen charging for the Pd/Co bilayer film is explained by different possible contributions.^76^ One of the proposed effects is the spintronic effect of spin pumping at the interface due to the Pd layer.^78^ Upon hydrogen absorption, the conductivity of the Pd layer decreases, which results in a reduction in the spin pumping contribution to the resonance linewidth of FMR. The second contribution is proposed to be due to the variation in the Gilbert damping by the variation in the d–d hybridization at the interface.^79,80^ The third effect is believed to be due to eddy current losses at the resonance linewidth upon the reduction in the conductivity of the Pd layer.^81,82^ It is further demonstrated that the nano-patterning of the Pd/Co/Pd multilayered films results in a higher sensitivity to hydrogen gas and a much faster desorption rate without any applied external magnetic field.^17^

**Fig. 11:**
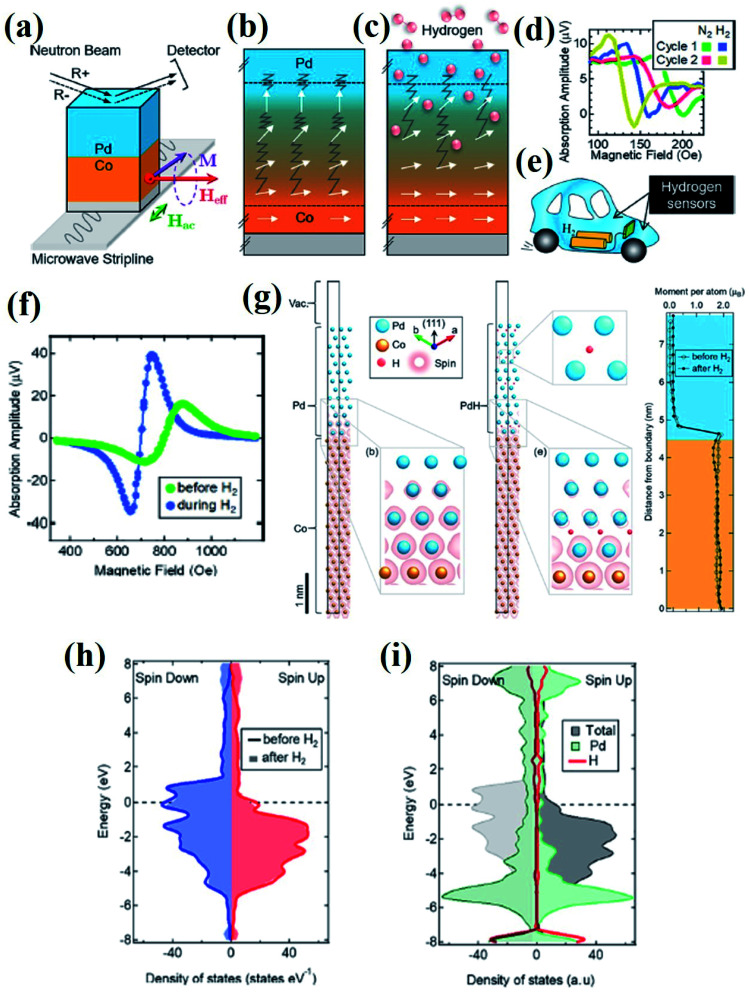
*In operando* study of the FMR based hydrogen sensing using Co/Pd film. (a) Polarized neutron reflectometry (PNR) with an *in situ* FMR experimental setup (b) before and (c) during exposure to H_2_ gas demonstrating the modification of Co/Pd interface with the change in the projection of the FM spins into the film plane. (d) FMR spectra of a Co (5 nm)/Pd (15 nm) film displaying excellent cycling ability between the first and second consecutive exposures to hydrogen gas. (e) Schematic illustration of the application of H_2_ sensor in hydrogen fuel cell vehicle. (f) Field-resolved FMR spectra of the Co/Pd film during hydrogen gas cycling. (g) DFT calculations of the theoretical Co/Pd thin-film structure. Electronic density of states determined from DFT calculations for a theoretical Co/Pd thin-film structure. (h) Total spin-resolved density of states and (i) site-projected density of states, including the projected density of states contributed by a hydrogen and a Pd site. Reprinted with permission from ref. 83. Copyright (2019) American Chemical Society.

Recently, Causer *et al.*^83^ reported the interface-resolved materials characterization of Co (5 nm)/Pd (8 nm) magnetic H_2_ gas sensors *in operando* during hydrogen gas cycling and revealed the physical mechanism. Combined observations from interface-sensitive polarized neutron reflectometry (PNR) with *in situ* FMR measurements and density functional theory (DFT) calculations revealed that the spin polarization at the Fermi level of the Co/Pd structure gets modified upon H_2_ gas absorption (Fig. 11). FMR experiments showed a broad spectrum that reached resonance at an applied field of *H*_res_ = 819 Oe, whereas H_2_ absorption changed the resonance position to a reduced field of *H*_res_ = 707 Oe. DFT calculations supported the experimental findings and indicated that an out-of-plane expansion of the Pd layer (∼7.5% increase in the overall thickness of the Pd layer upon exposure to H_2_) and electronic modification of the Co/Pd film occurs due to H_2_ absorption change in the total density of states and modified spin polarization at the Fermi level was observed, which provides insights to the magnetic H_2_ sensing based on FMR (Fig. 11g–i).

### 2.5 Magnetostatic surface spin-wave (MSSW) oscillator type magnetic gas sensors

#### 2.5.1 Overview, theories, and working principles

**Fig. 12:**
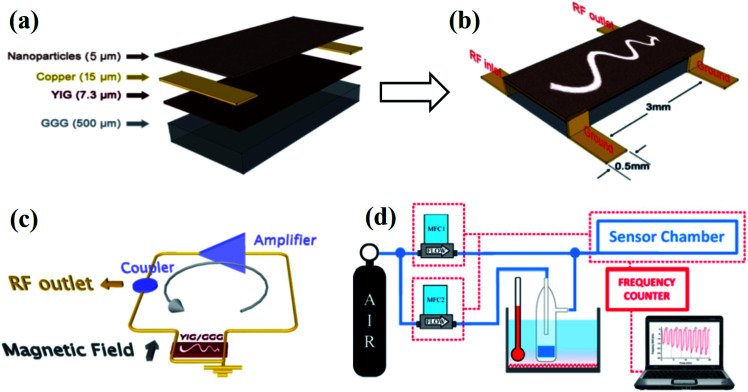
Two-port magnetostatic surface wave (MSSW) delay line for magnetic gas sensing. (a) Composition layer, (b) geometrical parameters, (c) schematic of the oscillator controller MSSW device, (d) schematic of the magnetic gas sensing instrumentation and set-up used for the measurements. Reprinted with permission from ref. 26. Copyright (2015) Royal Society of Chemistry.

Magnetostatic spin-wave (MSW)-based approaches along with the combination of a magnetic material as a sensitive layer are demonstrated to detect low concentrations of gases.^26,84^ Tunable MSW oscillators possess more than sufficient sensitivity to accurately measure weak variations induced in the magnetic characteristics of the sensitive material. YIG sphere oscillators based on the yttrium iron garnet (YIG) has been used in the MSSW instrument for the development of planar spin-wave technology.^85^ Based on these principles, Saniger and co-workers developed magnonic gas sensors based on magnetic (CuFe_2_O_4_) nanoparticles films coated on the YIG film.^22,84^ The fabrication details of the sensor devices are reported in their report, which is summarized in Fig. 12.^26^ The oscillation frequency (*f*) of the device can be approximated by

16 *f* = *f*_0_ + δ*F*_SL_ = *γ*(*H*_B_ + *H*_SL_) + *γ*δ*H*_SL_

where *f*_0_ is the unperturbated oscillation frequency, δ*F*_SL_ is the frequency shift due to the interaction between the sensitive magnetic layer, and the toxic gas, where *γ* = 2.8 MHz Oe^−1^ is the gyromagnetic constant, *H*_SL_ is the static magnetic field to be induced by the sensitive layer, and δ*H*_SL_ is the variation of the magnetic field induced by the interaction between the sensitive magnetic layer and toxic gas. The experimental set-up for MSSW-based gas sensing is shown in Fig. 12d.

#### 2.5.2 Recent developments

Based on the MSSW principles and CuFe_2_O_4_ nanoparticles as the magnetic layer, magnetic gas sensors have been developed.^26,84^ The sensor displayed good sensing performance in terms of the sensitivity, short response time, and good reproducibility, which could detect low concentrations of different volatile compounds such as dimethylformamide, isopropanol, and ethanol or aromatic hydrocarbons such as benzene, toluene, and xylene at room temperature.

**Fig. 13:**
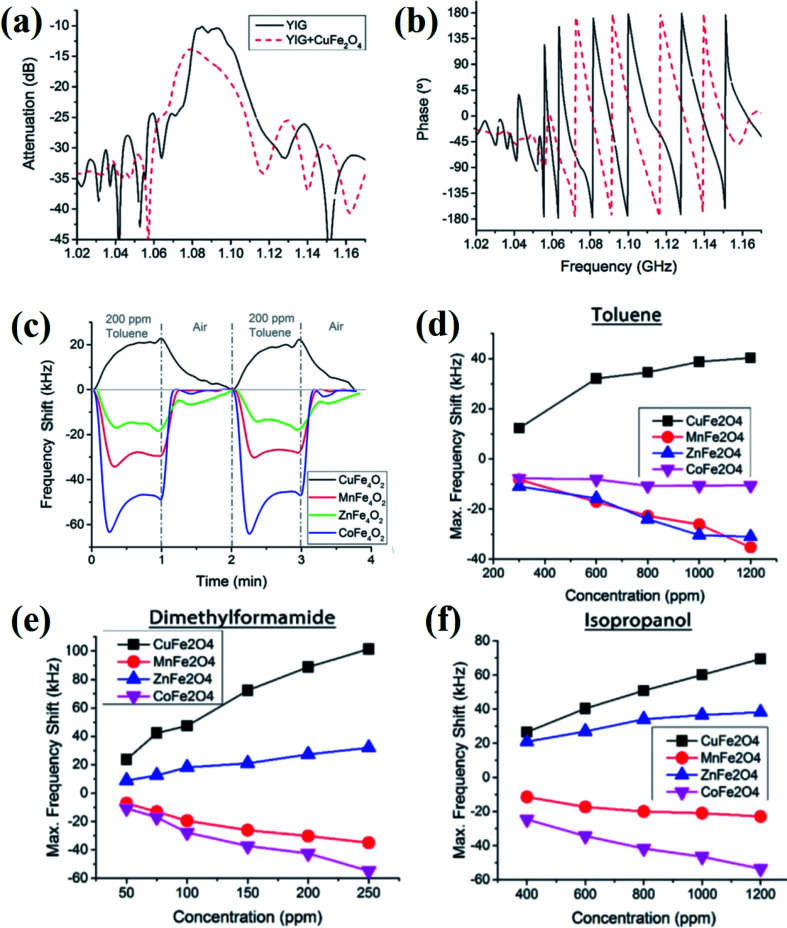
(a and b) Frequency response of the MSSW-based magnetic gas sensor device before and after being coated with CuFe_2_O_4_ nanoparticles. (c) Response of the magnetic gas sensors based on spinel oxides for 200 ppm toluene at room temperature. The response of the magnetic sensors at different concentrations of (d) toluene, (e) dimethylformamide, and (f) isopropanol. Reprinted with permission from ref. 84. Copyright (2017) Elsevier.

The observed frequency shifts in the presence of gas vapors were attributed due to the changes in the magnetic properties of the CuFe_2_O_4_ nanoparticles. Further, Matatagui *et al.*^84^ investigated the comparative magnetic gas sensing performance of magnetic nanoparticle layers of CuFe_2_O_4_, MnFe_2_O_4_, ZnFe_2_O_4_, and CoFe_2_O_4_ (Fig. 13). Typical frequency response curves in the presence of VOCs, dynamic response, and the response change in different concentrations of gas vapors are provided in Fig. 13. The applications of MSSW type sensors are mainly limited due to the complicated setup and high cost.^26,84^ The advantageous features of these emerging magnetic gas sensors along with their key limitations are summarized in Table 1.

**Table 1:** Advantages and disadvantages of different magnetic gas sensors

Type of sensor	Advantages	Disadvantages
Magnetization change	1. Highly sensitive to reducing or oxidizing gases	1. High cost
		2. Challenging set-up optimization
Hall effect	1. Weakly dependent on the analyte gas concentration in air	1. Sensitivity depends on the operating temperature
	2. High surface area of the nanostructures favors the enhanced sensing performance	2. Sensing response depend on the type (p- or n-type) of material
		3. Performance is highly dependent on the film composition, thickness, and field of operation
Kerr effect	1. High surface-sensitive properties	1. Tricky analyzer angle adjustment
	2. Signal is not affected by the paramagnetic and diamagnetic contributions of the substrates	
Ferromagnetic resonance (FMR) effect	1. Improve the signal to noise ratio	1. Proper adjustment of the magnetic field is needed
Magnetostatic surface spin wave oscillator (MSSW)	1. Detects low concentration of gases	1. High cost
	2. Accurately measures weak variations	2. Multistep, complicated experimental setup
	3. Good reproducibility	

## 3 Conclusion and future perspectives

We have reviewed the basic working principles, sensing mechanisms, and recent developments in magnetic gas sensing. Compared to their electrical property-based gas sensor counterparts, magnetic gas sensors have emerged as a more attractive candidate due to the following reasons. (i) No electrical contacts are needed to detect the gas, which lowers the risk of explosion due to fire when used in hydrogen-powered vehicles or in the presence of reactive chemicals or pollutants, (ii) the magnetic response is much faster compared to chemiresistive sensors, (iii) the working temperature of the sensors can be room temperature and it can be tuned to a very low or very high temperature by choosing magnetic materials with different Curie temperature (*T*_c_). We have provided a detailed description on the several effects used for magnetic gas sensing, which includes the Hall effect, Kerr effect, magnetization, spin change effects ferromagnetic resonance (FMR) effect, magneto-plasmonic effect, and magneto-static spin-wave (MSW) effect are employed for magnetic gas sensing applications. We have also reviewed recent developments on different materials used for magnetic sensing, which includes powder, thin films, and nanomaterials with magnetic (ferro- and antiferromagnets), diluted magnetic semiconducting (DMS) properties, and Pd alloys with transition metals (Co, Ni, Fe, Mn, and Cu). Subsequently, we have discussed the origin of enhanced sensitivity and working principles of magnetic gas sensors.

Although magnetic gas sensors based on different materials perform well, there is still a long way to go before it can be applied for further practical applications and there is plenty of room to investigate the gas sensing performances of several emerging advanced magnetic 2D materials. For example, black phosphorous, MXenes, and other groups of TMDs are not yet investigated for magnetic gas sensor device applications. Doping and modifying these 2D materials with magnetic materials can enable them to tune their magnetic properties for gas sensing applications. Tuning the number of layers and the modification of these 2D materials by different other approaches such as defect and vacancy engineering, alloying, intercalation, tuning the materials in *x*–*y* and *z* directions, and fabrication of heterostructures of 2D materials with a different orientation, by which the properties of the materials can be further tuned to achieve enhanced magnetic gas sensing performance. More investigations are needed to inspire efforts to address challenges such as a compatible and low-power operation in different interfering environments, stability, selectivity, speed, and multifunctionality. Piezotronic and piezophototronic effects are emerging areas in gas sensors research in recent years, which may be applied to magnetic gas sensor devices by choosing appropriate magnetic materials. Further, the fabrication of flexible, wearable, and self-powered gas sensor design is another important research area that needs to be explored in magnetic gas sensor devices. Further, research in this field by using active materials with high mechanical flexibility will create new dimensions and possibilities in the area of wearable magnetic gas sensor systems. In this regard, novel emerging materials in magnetic gas sensor device configuration and technologies need to be explored.

## Conflicts of interest

The authors declare no conflict of interest.

## Supplementary Material
